# Metastatic malignant melanoma in bone marrow with occult primary site – a case report with review of literature

**DOI:** 10.1186/1746-1596-2-38

**Published:** 2007-10-02

**Authors:** Deepali Jain, Tejindar singh, Naresh Kumar, Mradul K Daga

**Affiliations:** 1 Department of Pathology, Maulana Azad Medical College, New Delhi, India; 2 Department of Medicine, Maulana Azad Medical College, New Delhi, India.

## Abstract

**Background:**

Metastases of malignant melanoma to the bone marrow are very rare. A few case reports are published in the literature with a known primary site.

**Case presentation:**

Herein we present a case of metastatic malignant melanoma in bone marrow with occult primary site in a 22- year-old-male. Diagnosis was confirmed by morphology and immunohistochemistry. A pertinent review of literature is also presented by using relevant articles indexed in PubMed (National Library of Medicine) database. The search was based on the following terms: metastasis or metastases, malignant melanoma and bone marrow.

**Conclusion:**

In this report we discuss a rare case of metastatic malignant melanoma to the bone marrow with an unknown primary. Clinicians must be aware of the varied clinical manifestations of disseminated malignant melanoma even if the primary site is not evident.

## Background

Malignant melanoma accounts for 1–3% of all malignancies with an increasing incidence being seen worldwide [1]. Metastatic melanoma usually involves draining lymph nodes and occasionally adjacent skin first, but eventually metastasizes to distant visceral sites. The lung is most commonly involved followed by brain, liver, bone marrow, and intestine [2]. Metastases of melanoma to bone marrow are rare with widespread dissemination occurring in only 5–7% of cases [3]. In about 5–15% of cases, metastatic melanoma is detected in the absence of an identifiable primary tumor. In these cases it is generally believed that the primary tumor has regressed [4-6]. A search of the literature revealed many isolated case reports of metastatic melanoma to the bone marrow with a known primary [7]. However metastases to bone marrow with an occult primary have been reported rarely [8,9]. Herein we report a case of malignant melanoma metastasized to bone marrow with an occult primary site, along with a relevant review of literature. The case described is unique, as it describes an unusual clinical presentation of metastases, diagnosed only after bone marrow examination.

## Case presentation

**Figure 1:**
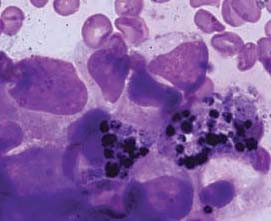
Bone marrow aspirate smear shows melanoma cells with nuclear pleomorphism and intracytoplasmic pigment, Leishman × 1000.

**Figure 2:**
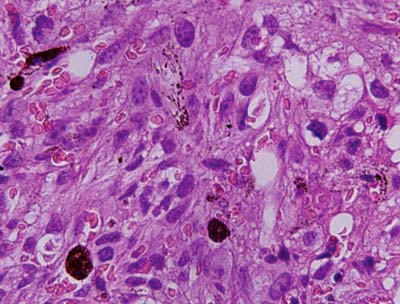
Bone marrow trephine biopsy shows spindle shaped malignant cells with dark brown granules of melanin pigment lying in the background of extensive fibrosis, H&E × 600.

**Figure 3:**
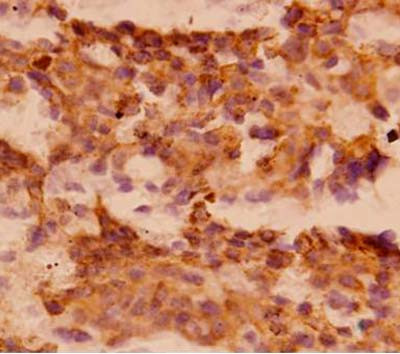
Photomicrograph shows strongly immunoreactive tumor cells for HMB-45 × 400.

A -22- year- old male presented with history of weakness, weight loss and right axillary swelling since two years and epistaxis and hemoptysis since one month. The swelling was progressively increasing for the past six months and did not respond to a three month course of antituberculous treatment given on the basis of fine needle aspiration cytology (FNAC), which revealed granulomatous inflammation and a positive Mantoux test. On examination patient was febrile, pale and ill- looking with enlarged right sided supraclavicular and inguinal lymph nodes. On palpation these lymph nodes were firm, discrete and mobile. A 10 × 10 cm soft, hot, tender, red and fluctuant irregular swelling was seen in the right axillary region. On incision and drainage it yielded about 200 ml of frank blood suggesting a hematoma. Cytological examination of the aspirate was not performed. Beneath the swelling, there was a single, firm, 1 × 1 cm, discrete lymph node, histopathology of which showed reactive hyperplasia. Fine needle aspiration cytology of other lymph nodes also revealed reactive morphology. Ultrasound examination of right axilla showed a large nodal mass measuring 7.3 × 12 cm seen in right lateral chest wall anterior to the pectoralis major. A mantoux tuberculin skin test (purified protein derivative, 5 tuberculin units) was positive with 21 mm of induration observed 48 hours after administration. Hematologic examination revealed severe anemia and thrombocytopenia. Hemogram findings were as follows: hemoglobin 5.5 gm/dL, white blood cells 7.8 × 10^9^/L, and platelets 65 × 10^9^/L. Peripheral blood film revealed a leucoerythroblastic blood picture. RBCs showed mild anisopokilocytosis. There were microcytes, tear drop cells and few nucleated RBCs (2/100WBCs). Mild degree of hypochromia was present. Platelets were reduced on blood smear. In the differential leucocyte count, neutrophils showed mild shift to the left- 2% myelocytes, 2% metamyelocytes, 46% polymorphs, 40%lymphocyte, 5% monocytes, and 5% eosinophils. Therefore, the overall picture of leukoerythroblastosis was considered. Patient did not respond to hematinics and his cytopenias worsened over time. Despite 15 (5 packed cells, 4 platelets, 6 fresh frozen plasma) transfusions with in a period of 10 days the patient's condition progressively deteriorated. Bone marrow aspiration yielded no particles but the trails were cellular and showed replacement of normal marrow elements by scattered and a few small cohesive clusters of large abnormal cells having round to mildly irregular nuclei with coarse chromatin, one to two prominent nucleoli and abundant amount of cytoplasm. A few cells showed cytoplasmic vacuolations. On close inspection some cells and a few background histiocytes showed brownish cytoplasmic pigmentation (Figure 1). Occasional interspersed erythroid and myeloid precursors were present, but no megakaryocytes were seen. Bone marrow iron was grade 1. A tentative report of bone marrow metastases was conveyed. Trephine biopsy showed depression of normal hematopoiesis by a diffuse infiltrate of pleomorphic, loosely cohesive malignant cells having hyperchromatic nuclei and distinct nucleoli. Cytoplasmic pigment was present in some tumor cells (Figure 2) and histiocytes. Coarsening of reticulin fibers was seen. Immunohistochemical staining on trephine biopsy showed that the tumor cells were negative for cytokeratin, but positive for S-100 protein and HMB-45 (Figure 3). Finally a definitive report of bone marrow metastases of malignant melanoma was given with advice to search for the primary site. Computed tomography (CT) and chest x-ray were normal. CT and abdominal ultrasound were also within normal limits. While investigating patient, no previous history of a skin lesion or surgery was elicited. There was no past exposure to UV light or organic dyes. Lastly, no primary site could be identified and patient was referred to a tertiary centre for chemotherapy.

## Discussion

Epithelial tumors of the thyroid, breast, lung, kidney and prostate commonly metastasize to the marrow [10]. However bone marrow infiltration of human melanoma is found in only 7% of in vivo staging procedures and in up to 45% of autopsy cases [11]. There are a number of reports of malignant melanoma showing infiltration of marrow [see Additional File 1] [3,7-10,12-29]. The male female ratio in reviewed cases was 1.33:1. Age ranged from 3 to 75 years (median 60; mean 51.5 years). Anemia was the most common hematologic manifestation (7 of 21 patients) followed by thrombocytopenia, pancytopenia and leukoerythroblastosis. One patient had carcinocythemia at the time of presentation. Most of the reviewed cases died due to the disseminated disease despite chemotherapy. Savage et al [3] studied 97 patients of malignant melanoma, of these, 5.4% had marrow involvement. Bone marrow involvement from retroperitoneal, anal, tonsillar, nasal, osseous and ocular melanoma has been described [18,24,25,28,29]. Interestingly, Spiller et al reported a case of giant congenital nevus with metastatic malignant melanoma in a child [27]. There was no identifiable change in the nevus to suggest the primary site for malignant transformation. The Metastatic involvement of bone marrow by melanoma with an occult primary has been reported rarely [8,9]. In one such case, history of intense childhood exposure to ultraviolet light and an occupational exposure to organic dyes was present [9]. However we did not find past history of such exposures in the index patient. At initial presentation our patient had a two year history of weakness and right axillary swelling. The hemogram showed bicytopenia and leukoerythroblastosis. Extensive bone marrow involvement normally presents with pancytopenia but surprisingly our patient had normal white blood cell count with a left shift. Extreme leukocytosis in a case of metastatic melanoma has been reported by de Wolff et al [26]. It was attributed to either bone marrow invasion by metastases or due to production of cytokines or other mediators by tumor cells. In contrast, autoimmune neutropenia and thrombocytopenia have been reported in association with malignant melanoma as a rare paraneoplastic phenomenon [19,30]. Interestingly Bhagwati et al [9] described microangiopathic hemolytic anemia and disseminated intravascular coagulation related to widely disseminated melanoma. The patient's lymph nodes were unremarkable on histopathology presumably due to hematogenous dissemination of the tumor. This in itself lends to a very poor prognosis [31]. Frank blood was obtained on surgical drainage of axillary swelling. Presumably, axillary hematoma might have been the site of lymph nodal metastases which was not revealed as pathologic examination was not performed on drained fluid. The simultaneous presentation of axillary node metastases and bone marrow involvement is exceptionally rare [11,19]. The primary site might have regressed over the period of time in the index patient. Partial regression is a common feature in melanoma. Total regression is much less common, but numerous cases in which the primary tumor regressed completely after giving rise to nodal and distant metastases have been documented. In about 5–15% of the patients who present initially with metastases of melanoma, no primary tumor can be found [4-6]. Although the primary tumor may in some instances be in an internal organ, it was in most instances located in the skin and regressed spontaneously [32]. Parenthetically, the prognosis of patients with metastatic malignant melanoma and an unknown (presumably regressed) primary is the same as for the patients with an overt primary malignancy [6]. Malignant melanoma involves bone marrow with disseminated disease and is often amelanotic [20]. However while reviewing literature, we found only three cases of amelanotic melanoma [20,21,26]. If melanin is found in the tumor cells or macrophages as brown pigment, the diagnosis is relatively straightforward similar to the index case. On the other hand, differential diagnoses of amelanotic melanoma would be other solid tumors such as lymphomas, carcinomas and metastatic small round cell tumors. The primary site of the malignant deposits may be extremely difficult to determine on a morphological basis alone, but their origin can sometimes be inferred from their morphological appearance, especially in mucous producing carcinoma, squamous carcinoma, some adenocarcinomas, and in many cases of metastatic neuroblastoma. Melanoma should be suspected if the tumor comprises of histologically polygonal or spindle cells with prominent nucleoli. Immunohistochemical study is useful in supporting a diagnosis of malignant melanoma. Typically melanoma is reactive for vimentin, S-100 protein, HMB-45, melan -A, tyrosinase, and microphthalmia transcription factor [33]. HMB-45 is a much more specific marker than S-100 protein [34]. Our case is in fact entirely typical of malignant melanoma based on morphology and immunohistochemistry.

## Conclusion

Despite the rarity of bone marrow involvement, clinicians must be aware of the varied clinical manifestations of disseminated malignant melanoma even if the primary site is not evident.

## Competing interests

The author(s) declare that they have no competing interests.

## Authors' contributions

DJ is primarily responsible for drafting, literature search, and submission of the manuscript. TS assisted in reviewing the slides. NK and MKD supervised treatment. All authors have read and approved the final manuscript.

## Supplementary Material

Additional file 1 Table 1. Review of literature of previously reported cases of malignant melanoma metastasized to bone marrow. Click here for file
