# Supplementary material for: Metastatic malignant melanoma in bone marrow with occult primary site – a case report with review of literature
Source: Diagn Pathol. 2007 Oct 2;2:38. doi: 10.1186/1746-1596-2-38 (PMC2140052; doi:10.1186/1746-1596-2-38)
Supplement: Additional file 1 — Table 1. Review of literature of previously reported cases of malignant melanoma metastasized to bone marrow. [file 1746-1596-2-38-S1.doc]

**Table 1:** Review of literature of previously reported cases of malignant melanoma metastasized to bone marrow

| S.N | Author | Year | No of cases | Age/ sex | Primary site | Clinical features | Associated features | Treatment |
| --- | --- | --- | --- | --- | --- | --- | --- | --- |
| 1 | Battle and Stasney[12] | 1941 | 1 | 60/F | Rt. Eye | Severe back pain and weight loss. | Anemia | NA |
| 2 | Rubinstein MA et al [13] | 1948 | 1 | 47/F | NA | Pain in the right leg, weight loss and a mass in the lower abdomen. X-ray showed some destruction of the lumbar spine. | Anemia | NA |
| 3 | Jonsson and Rundles [14] | 1951 | 1 | NA | NA | NA | NA | NA |
| 4 | Durkee and Wilson [15] | 1951 | 1 | 48/M | Lt Eye | NA | NA | NA |
| 5 | Franklin JW et al [16] | 1952 | 2 | 67/M 18/M | 1. Wart” on Interscapular area  2. Left neck | 1. Severe back pain, weakness, anorexia and wt loss 2. weakness, vomiting and short ness of breath | 1. Anemia | NA |
| 6 | Motulsky and Rohn[17] | 1953 | 4 | NA | NA | NA | NA | NA |
| 7 | Savage RA et al [3] | 1983 | 5 | NA | NA | NA | NA | NA |
| 8 | Gallivan MVE et al [18] | 1984 | 1 | 55/F | Retroperitoneal | Abdominal pain | Carcinocythemia | Died |
| 9 | Brown D et al [7] | 1990 | 1 | 66/M | Iris left eye | NA | Bony lesions and a mass in the liver | Died |
| 10 | Basile M et al [19] | 1992 | 1 | 63/M | Skin lesion? | Lt. Axillary mass | Simultaneous lymph node involvement, Immune-mediated thrombocytopenia, spontaneous regression? | Chemotherapy, patient died 6 months after diagnosis |
| 11 | Tuncer I et al [8] | 1994 | 1 | 14/F | Unknown | NA | NA | NA |
| 12 | Villarrubia J et al [20] | 1995 | 1 | NA | NA | NA | Amelanotic melanoma | NA |
| 13 | Bhagwati N et al [9] | 1998 | 1 | 34/M | Unknown | NA | Microangiopathic hemolytic anemia, and disseminated intravascular coagulation | Died 3 weeks after diagnosis |
| 14 | Trefzer U et al [21] | 1999 | 1 | 48/M | NA | Fatigue, weakness, weight loss, shortness of breath, and limb pain | Thrombocytopenia, anemia, marked leucocytosisFulminant Intravascular Disseminated Amelanotic melanoma | Died |
| 15 | Invernizzi R et al [22] | 2001 | 1 | 34/M | Rt. Ear | Fever, hepatosplenomegaly (HSM) | Thrombocytopenia, anemia, WBC normal | NA |
| 16 | Chim CS et al [23] | 2001 | 1 | 67/M | Ocular | Painful Rt. eye with blurring of vision, HSM | Leucoerythroblastic Picture | NA |
| 17 | Basu D et al [24] | 2002 | 2 | 74/M35/M | AnalLeft tonsil | 1. Generalized weakness and pallor 2.Paraparesis and urinary retention | 1. Leucoerythroblastic Picture2. X-ray: T 12 Collapse | NA |
| 18 | Sechadendrof D et al [25] | 2003 | 1 | 70/F | Ocular | Nocturnal sweating and increasing epigastric pain | NA | NA |
| 19 | de Wolff JF et al [26] | 2004 | 1 | 63/F | Lt. Ankle | Progressive fatigue, breathlessnesssplenomegaly | Extreme leucocytosis, anemia,Amelanotic melanoma | Died |
| 20 | Spiller SE et al [27] | 2005 | 1 | 3/M | Unknown | Intermittent limping back pain, episodes of brown urine, periorbital edema | Pancytopenia, Giant nevus extending from scalp to back and chest | Chemotherapy, died |
| 21 | Uesawa M et al [28] | 2006 | 1 | 67/F | Right maxillary bone | Lower back pain | NA | NA |
| 22 | Batsis and Barry [10] | 2006 | 1 | 75/F | Neck | Progressive right sided  weakness, vomiting, constipation, decreased appetite  and central abdominal discomfort | Mild leucocytosis, Hypercalcaemia | Died 3 weeks |
| 23 | Wong KF [29] | 2006 | 1 | 74/F | Rt. nasal cavity | NA | Pancytopenia | NA |
| 24 | Present case | 2007 | 1 | 22/M | Unknown | Weakness right axillary swelling, hemoptysis and epistaxis | Anemia with thrombocytopenia | Died |

Abbreviations- NA: not available, M: male, F:female, Rt: right, Lt: left
